# Aromatherapy With Citrus Aurantium Oil and Anxiety During the First Stage of Labor

**DOI:** 10.5812/ircmj.18371

**Published:** 2014-06-05

**Authors:** Masoumeh Namazi, Seddigheh Amir Ali Akbari, Faraz Mojab, Atefe Talebi, Hamid Alavi Majd, Sharareh Jannesari

**Affiliations:** 1 Department of Midwifery, Faculty of Nursing and Midwifery, Shahid Beheshti University of Medical Sciences, Tehran, IR Iran; 2 Department of Traditional Pharmacy, Shahid Beheshti University of Medical Sciences, Tehran, IR Iran; 3 Department of Biostatistics, Faculty of Paramedicine, Shahid Beheshti University of Medical Sciences, Tehran, IR Iran

**Keywords:** Citrus aurantium, Anxiety, Complementary Medicine, Medicinal Plants, Aromatherapy

## Abstract

**Background:**

Anxiety is the most common psychological response of women to labor. Aromatherapy, i.e. the use of fragrant essential oils to stimulate the olfactory system, can create a state of calmness and help to alleviate anxiety.

**Objectives:**

The present study tried to determine the efficacy of aromatherapy with *Citrus aurantium* oil in reducing anxiety during the first stage of labor.

**Patients and Methods:**

This randomized clinical trial was conducted on two groups of pregnant women, referred to Vali-Asr Hospital (Tuyserkan, Iran) between June and September 2013. The sample size was comprised of 63 subjects in each group. Gauzes impregnated with 4 mL of *C. aurantium* distillate and normal saline were attached to the collar of subjects in the aromatherapy and control groups, respectively. The gauzes were changed every 30 minutes. The levels of anxiety in both groups were measured at baseline and after the intervention at dilations of 3-4 and 6-8 cm. The participants were followed up until delivery and the first- and fifth-minute Apgar scores were recorded. Data were collected using a demographic and obstetric characteristics questionnaire, an examination and observation checklist, and Spielberger state-trait anxiety questionnaire. Data analysis was performed with independent-t, Mann-Whitney, and chi-square tests in SPSS-22. P values less than 0.05 were considered significant.

**Results:**

Before the intervention, both groups had same levels of anxiety. However, the levels of anxiety at dilations of 3-4 and 6-8 cm were significantly lower in the aromatherapy group compared with the control group.

**Conclusions:**

The results of this study confirmed aromatherapy with *C. aurantium* blossom oil as a simple, inexpensive, noninvasive, and effective intervention to reduce anxiety during labor.

## 1. Background

Anxiety is the most common psychological response of women to labor (1). In fact, 80% of women in labor have anxiety disorders (2). According to the control theory, there is a relationship between pain and psychological problems like anxiety. Women with lower levels of anxiety experience less pain during labor. In other words, in the presence of anxiety, severe spasm of the pelvic floor and perineal muscles cause increased labor pain (3). Anxiety and stress during labor may decrease the amplitude and frequency of uterine contractions and thus, increase the labor duration and the likelihood of assisted delivery and even cesarean section. Moreover, more bleeding during labor and delayed onset of lactation have been observed among anxious women (1). Accordingly, various measures including constant support during delivery (4), relaxation and breathing techniques, music therapy (5), bathing or showering, and complementary medicine are currently taken to reduce women’s stress during labor (6). Complementary medicine has received particular attention as a modern method of care for women during prenatal, perinatal, and postnatal periods (7). Aromatherapy, a form of complementary medicine, seeks to reduce stress and induce a feeling of calmness by stimulating the olfactory system through the use of essential oils (8). Leite et al. reported antianxiety effects of inhaling the essential oil of *Citrus aurantium* (bitter orange) in mice (9). Lehrner et al. found that inhalation of lavender and orange essences had the mentioned effects on patients attending dental offices (10). Valipour et al. showed that aromatherapy with rose essence reduced anxiety levels of 120 primiparous women at labor (8). Ozgoli et al. suggested the efficacy of inhaling peppermint essence in soothing the pain and anxiety of primiparous women through the first stage of labor (11). Likewise, Tafazoli et al. introduced lavender essence to decrease anxiety during labor (12).

Essential oil of *C. aurantium* is widely used in aromatherapy. Besides, neroli oil, a strongly scented bitter liquid, is produced from water distillation of freshly gathered blossoms of *C. aurantium* or orange tree. The oil is amber-colored, but turns red in light (13-15). According to the available literature, essential oil of *C. aurantium* stimulates the central nervous system, enhances the mood, lowers blood pressure, and has sedative, analgesic, anti-inflammatory, antispasmodic, carminative, digestive, and diuretic effects. Clinical trials have also reported it to have antidepressant effects, similar to that of fluoxetine (16-18). Flavonoids are found in most natural compounds. They are necessary for body cells of vertebrates (19) and have numerous pharmacological properties. Since they inhibit the oxidation of low-molecular weight proteins and platelet accumulation and contribute to immune cell stability, they have applications in treatment of mental disorders, viral infections, inflammation, and allergies (20). Furthermore, flavonoids act as benzodiazepine receptor agonists and can thus reduce anxiety (21).

## 2. Objectives

Despite the importance and benefits of reducing anxiety during labor, no previous Iranian studies have evaluated the efficacy of *C. aurantium* essential oil. Therefore, the present research assessed effects of the mentioned oil on anxiety during labor.

## 3. Patients and Methods

This randomized clinical trial was conducted on two groups of pregnant women, referred to Vali-Asr Hospital (Tuyserkan, Iran) between June and September 2013. It was registered at the Iranian Registry of Clinical Trials (IRCT ID: N6 201301306807). Considering similar studies, confidence interval of 95%, and probability of error of 5%, the sample size was calculated as 63 subjects in each group. Women were first briefed about the objectives and methods of study and then asked to provide written consent if they were willing to participate. The women were only included if they were Iranian, primiparous, and 18-35 years old, had term, singleton pregnancy, cephalic presentation, spontaneous contractions, 3-4 cm cervical dilation at the onset of labor, good hip condition and intact amniotic sac, had not taken analgesic drugs in the past eight hours, and did not have any known liver, gallbladder, or respiratory diseases on their records. Individuals with smell disorders, taking allergy to herbal medicines, or having pregnancy complications (e.g. preeclampsia, chorioamnionitis, placental abruption, and abnormal fetal heart rate at the time of study) were not included. Data were collected using a demographic and obstetric questionnaire, an examination and observation checklist including vital signs, vaginal examination, uterine contractions, and fetal heart rate, and Spielberger state-trait anxiety questionnaire. Validities of the first two tools were tested through content validity. Moreover, reliability of the checklist was approved using the parallel form reliability (r = 0.85). After completing the first two instruments, severity of the state anxiety was assessed with 20 items of the mentioned anxiety questionnaire. Since each item was scored as 1-4, the total anxiety score ranged between 20 and 80 (20-40, mild anxiety; 41-60, moderate anxiety; 61-80, severe anxiety). This questionnaire is widely used to measure state-trait anxiety in clinical studies (11, 22-24) and has a correlation coefficient of 0.85-0.91 (25).

**Figure 1.**
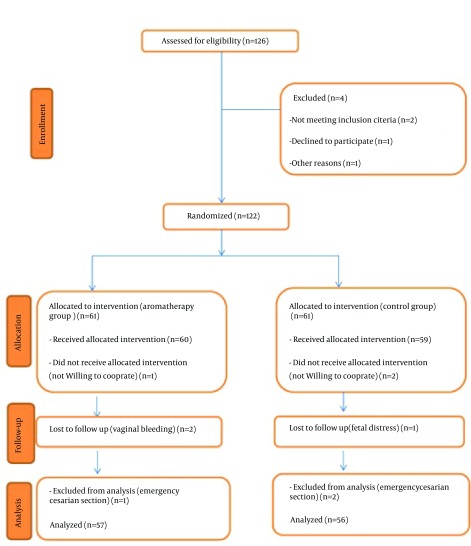
Consort Flowchart of the Study

The exclusion criteria were *C. aurantium* allergy or intolerance, pregnancy complications such as vaginal bleeding during the study, and emergency cesarean before completion of the study. A table of random numbers was used to randomly allocate the eligible women to aromatherapy and control groups (n = 63 each). Concentration of the purchased *C. aurantium* distillate (Iran-Gereban Co., Iran) was determined by gravimetric method at the School of Pharmacy, Shahid Beheshti University of Medical Sciences (Tehran, Iran). Each 100 mL of the distillate contained 8 mL *C. aurantium* essential oil. Gauzes impregnated with 4 mL of *C. aurantium *distillate and normal saline were attached to the collar of the participants in the aromatherapy and control groups, respectively. The gauzes were changed every 30 minutes. Intensity of anxiety in both groups was measured at baseline and after the intervention at dilations of 3-4 and 6-8 cm. Data analyses were performed with SPSS for Windows version 22.0 (SPSS Inc., Chicago, IL, USA). Descriptive statistics including central tendency, dispersion, and frequency distribution were used to describe the two groups. Chi-square and Fisher’s exact tests were applied to compare the qualitative variables between the groups. Quantitative variables were compared using independent t-tests. Comparisons of the two groups in terms of qualitative variables with non-normal distribution and ordinal variables were made using nonparametric Mann-Whitney test. Finally, Kolmogorov-Smirnov test was conducted to examine normal distribution of the subjects (Figure 1).

## 4. Results

This study was conducted on 126 primiparous women in two groups of aromatherapy (n = 63) and control (n = 63). The two groups had no significant differences regarding individual, social, or obstetric characteristics such as age, education, occupation, gestation age, and frequency and amplitude of uterine contractions (Tables 1 and 2). Before the intervention, both groups had the same levels of anxiety; however, the levels of anxiety at dilations of 3-4 and 6-8 cm were significantly lower in the aromatherapy group compared with the control group (Table 3).

**Table 1.** Individual and Social Characteristics of Primiparous Women Admitted to Vali-Asr Hospital ^a^

Variable	Aromatherapy Group	Control Group	P Value
**Mothers' age, y**	26.43 ± 3.216	26.60 ± 3.406	0.768
**Education**			0.967
High school and lower	79.4	82.5	
University degree	20.6	17.5	
**Pregnancy age, wk**	38.30 ± 0.978	38.08 ± 1.067	0.225
**Profession**			0.650
Housewife	79.4	82.5	
Working	20.6	17.5	
**Wanted pregnancy**	79.4	82.5	0.650

^a^ Data are presented as mean ± SD or %.

**Table 2.** Obstetric Characteristics of Primiparous Women Admitted to Vali-Asr Hospital ^a^

Variable	Aromatherapy Group	Control Group	P Value
**Contraction length in 3-4 cm dilatation**	44.08 ± 0.703	43.94 ± 0.759	0.275
**Contraction length in 5-7 cm dilatation**	47.40 ± 0.493	47.27 ± 0.447	0.133
**Contraction length in 8-10 cm dilatation**	49.44 ± 0.501	49.43 ± 0.499	0.859
**Contraction frequency during 10 min in 3-4 cm dilatation**	2.27 ± 0.447	2.27 ± 0.447	> 0.999
**Contraction frequency during 10 min in 5-7 cm dilatation**	3.16 ± 0.368	3.22 ± 0.419	0.368
**Contraction frequency during 10 min in 8-10 cm dilatation**	3.81 ± 0.396	3.73 ± 0.447	0.294

^a^ Data are presented as Mean ± SD.

**Table 3.** Anxiety Scores of Primiparous Women Admitted to Vali-Asr Hospital at Different Dilations^a^

Dilatation Stages	Aromatherapy Group	Control Group	P Value
**Before intervention**	55.16 ± 1.247	61.86 ± 1.327	0.403
**3-4 cm dilatation**	45.32 ± 1.216	56.38 ± 1.128	< 0.001
**6-8 cm dilatation**	43.19 ± 1.664	59.32 ± 1.584	< 0.001

^a^ Data are presented as Mean ± SD.

## 5. Discussion

Anxiety scores of the two groups in the present study showed that aromatherapy with *C. aurantium* could reduce anxiety during labor. According to Bastard et al. and Smith et al. essential oils improved mood and reduced anxiety during labor by stimulating the olfactory pathways in the limbic system (26, 27). Essential oils are absorbed through inhalation, affect enzymes and ion canals and receptors, and eventually stimulate the brain. They can hence relieve anxiety, have antidepressant effects, and increase the blood circulation in the brain. Entering the body through inhalation also enables these oils to cross the blood-brain barrier and interact with central nervous system receptors (28). Aromatherapy with essential oil of *C. aurantium* significantly reduced anxiety in mice (9, 15). In a study comparing the effects of aromatherapy with *C. aurantium* and diazepam on preoperative anxiety levels, Akhlaghi et al. measured patients’ anxiety levels before and two hours after the intervention. They observed that anxiety scores (according to Spielberger state-trait anxiety questionnaire) reduced in the aromatherapy group and diazepam group. The significant difference between the two groups after the intervention suggested the efficacy of aromatherapy with *C. aurantium* in reducing preoperative anxiety (21). While Akhlagi et al. assessed both state and trait anxieties only once after the intervention and administered diazepam in the control group, we measured state anxiety at two stages after the intervention and used normal saline as the placebo. Imura et al. investigated the mental-psychological effects of 30-minute aromatherapy massage (with lavender and *C. aurantium* oils) on postpartum women. They found that compared to the routine care, the intervention could boost the subjects’ moods (6). However, unlike the present study, Imura et al. investigated not only anxiety, but also other variables such as depression, fatigue, and anger. On the other hand, they carried out the intervention only once and did not use a placebo in the control group. Furthermore, the observed difference in the anxiety score could have been caused by the combination of massage and aromatherapy and cannot be attributed to aromatherapy alone.

Considering the absence of other studies on the effect of aromatherapy with *C. aurantium* on anxiety, we compared our findings with those of similar studies, using the essential oil of orange (with compounds comparable to *C. aurantium* compounds) (13). Lehner et al. reported that aromatherapy with orange oil significantly reduced the mean state anxiety in female patients undergoing dental surgeries (10). Although Lehner et al. used the same instrument as ours to measure state anxiety, they did not mention the duration of exposure to the oil. In another study, Lehner et al. compared the effects of lavender and orange oils and music therapy on anxiety levels of patients attending dental offices. They indicated that state anxiety of individuals who received aromatherapy with orange oil was 19.4% lower than that of the control group (29). In contrast with our findings, in a comparison between the efficacy of music therapy and aromatherapy alone and in combination, Holm and Fitzmaurice suggested that inhalation of orange oil had no significant effect on the anxiety levels of children admitted to emergency wards. They justified such finding by inappropriate application of the oil; i.e. they used an electric diffuser to disperse the oil, but most of the patients failed to sense the aroma. Therefore, the researchers concluded that a different diffuser or higher amount of orange oil might have been required. Besides, the air-conditioning system of the hospital could have decreased the strength of the aroma. Apparently, aromatherapy could not be successful under such conditions (2). Finally, as we did not detect any significant differences between the two groups in terms of the mean first- and fifth-minute Apgar scores, aromatherapy with *C. aurantium* did not seem to have any adverse effects on the fetus. Considering the limited number of studies on the effects of aromatherapy with *C. aurantium* oil on anxiety, future studies are recommended to evaluate the efficacy of *C. aurantium* oil in reducing anxiety and the probable biochemical mechanisms involved. The current study confirmed aromatherapy with *C. aurantium* blossom oil as a simple, inexpensive, and noninvasive intervention to reduce anxiety during labor. We found no studies related to the effect of *C. aurantium* on anxiety during the first stage of labor or even anxiolytic effect of this herbal medicine in human in the literature review. Only a few studies reported that some similar components of *Citrus* may reduce anxiety. Our study demonstrated that *C. aurantium *may reduce childbirth anxiety. This result could trigger the use of herbal medicine during labor.
